# Identification and bioinformatic analysis of neprilysin and neprilysin-like metalloendopeptidases in *Drosophila melanogaster*

**DOI:** 10.17912/micropub.biology.000410

**Published:** 2021-06-23

**Authors:** Heiko Meyer, Annika Buhr, Patrick Callaerts, Ronja Schiemann, Mariana F. Wolfner, Steven J. Marygold

**Affiliations:** 1 Department of Zoology & Developmental Biology, Osnabrück University, 49076 Osnabrück, Germany; 2 Laboratory of Behavioral and Developmental Genetics, Department of Human Genetics, KULeuven, University of Leuven, B-3000 Leuven, Belgium; 3 Department of Molecular Biology & Genetics, Cornell University, Ithaca NY 14853 USA; 4 FlyBase, Department of Physiology, Development and Neuroscience, University of Cambridge, Cambridge, CB2 3DY, U.K.

## Abstract

The neprilysin (M13) family of metalloendopeptidases comprises highly conserved ectoenzymes that cleave and thereby inactivate many physiologically relevant peptides in the extracellular space. Impaired neprilysin activity is associated with numerous human diseases. Here, we present a comprehensive list and classification of M13 family members in *Drosophila melanogaster*. Seven *Neprilysin* (*Nep*) genes encode active peptidases, while 21 *Neprilysin-like* (*Nepl*) genes encode proteins predicted to be catalytically inactive. RNAseq data demonstrate that all 28 genes are expressed during development, often in a tissue-specific pattern. Most Nep proteins possess a transmembrane domain, whereas almost all Nepl proteins are predicted to be secreted.

**Table 1. Genes encoding Drosophila melanogaster neprilysins and neprilysin-like metalloendopeptidases.**
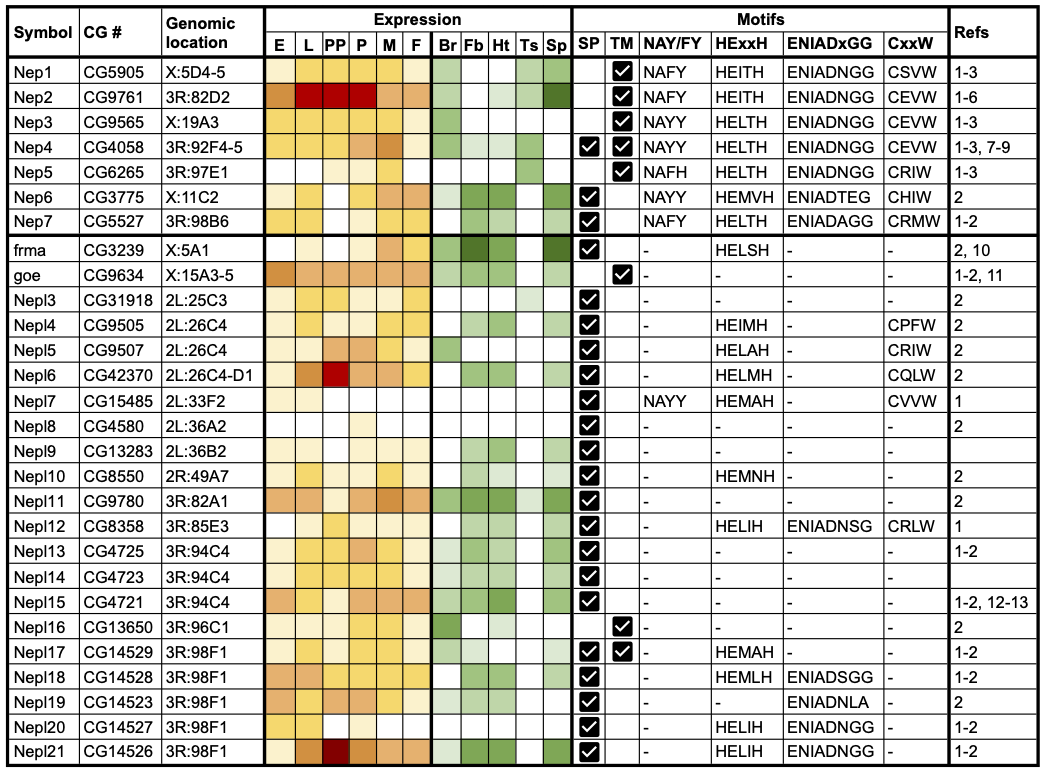
Symbol: symbol of the *Drosophila* gene in FlyBase; CG #: gene model annotation ID; Genomic location: chromosome scaffold and FlyBase-computed cytological location of the gene; Expression: heat-map representation of expression levels throughout development (yellow/red scale; embryos (E), larvae (L), prepupae (PP), pupae (P), adult males (M) and adult females (F)) and in adult tissues (green scale; brain/CNS (Br), fat body (Fb), heart (Ht), testis (Ts) and spermatheca (Sp)), where white represents undetectable expression; Motifs: presence of a signal peptide (SP) or a single transmembrane domain (TM) is indicated, together with the amino acid sequence (if present) of each of the four conserved sequence motifs required for catalytic activity; Refs: reference(s) identifying/characterizing the *Drosophila* gene/protein: 1) Bland *et al.,* 2008, 2) Sitnik *et al.,* 2014, 3) Meyer *et al.,* 2011, 4) Thomas *et al.,* 2005, 5) Bland *et al.,* 2007, 6) Bland *et al.,* 2009, 7) Meyer *et al.,* 2009, 8) Panz *et al.,* 2012, 9) Hallier *et al.,* 2016, 10) Findlay *et al.,* 2014, 11) Matsuoka *et al.,* 2014, 12) Nfonsam *et al.,* 2012, 13) Banerjee *et al.,* 2021.

## Description

Neprilysins belong to the family of M13 metalloendopeptidases and constitute highly conserved ectoenzymes that cleave and thereby inactivate numerous physiologically relevant peptides in the extracellular space. While the vast majority of these enzymes appear to be membrane-bound, some family members have been identified as soluble secreted proteins (Turner *et al.,* 2001). In humans, seven members of the M13 family are known, namely Neprilysin (NEP), endothelin-converting enzymes (ECE1, ECE2), ECEL1, MMEL1, the KELL blood-group protein and PHEX (Turner and Tanzawa 1997). Among these, NEP is characterized best, with identified substrates including endothelins, angiotensins I and II, enkephalins, bradykinin, atrial natriuretic peptide, substance P and the amyloid-beta peptide (Turner *et al.,* 2001, Nalivaeva *et al.,* 2020). NEP-mediated hydrolysis is critical to maintain the physiological homeostasis of these peptides, and thus represents a prerequisite for proper endocrine signal transmission. Accordingly, impaired neprilysin activity in humans is involved in the pathogenesis of numerous diseases, including hypertension (Molinaro *et al.,* 2002), analgesia (Whitworth 2003), cancer (Turner *et al.,* 2001) and Alzheimer’s disease (Iwata *et al.,* 2000, Belyaev *et al.,* 2009). Clinical trials have confirmed the therapeutic potential of modulating neprilysin activity (Jessup 2014).

Previous analyses of the *Drosophila melanogaster* (hereafter, *‘Drosophila’*) genome have identified up to 24 genes encoding M13 family members (Coates *et al.,* 2000, Isaac *et al.,* 2000, Bland *et al.,* 2008, Sitnik *et al.,* 2014). Five of these (*Nep1*–*Nep5*) are thought to encode catalytically active neprilysins. However, this has so far been demonstrated only for Nep2 and Nep4 (Thomas *et al.,* 2005, Bland *et al.,* 2007, Meyer *et al.,* 2009, Hallier *et al.,* 2016), and *in vivo* substrates are known only for Nep4 (Hallier *et al.,* 2016). Nep2 is involved in the regulation of locomotion and geotactic behavior (Bland *et al.,* 2009) and is required for early embryonic development (Sitnik *et al.,* 2014). Nep4 is implicated in sustaining muscle integrity (Panz *et al.,* 2012) and controls insulin signaling and feeding behavior (Hallier *et al.,* 2016). Neprilysin activity in general appears to be critical to the formation of middle- and long-term memory (Turrel *et al.,* 2016), reproduction (Sitnik *et al.,* 2014) and regulation of pigment dispersing factor signaling within circadian neural circuits (Isaac *et al.,* 2007). Significantly, increased expression of *Nep1* or *Nep2* ameliorates the detrimental effects of amyloid-beta peptide overexpression in *Drosophila* models of Alzheimer’s disease (Finelli *et al.,* 2004, Cao *et al.,* 2008, Sofola-Adesakin *et al.,* 2016, Turrel *et al.,* 2017, Turrel *et al.,* 2020). Other *Drosophila* M13 members lack key catalytic residues and are therefore predicted to be inactive or have non-enzymatic functions (Bland *et al.,* 2008, Sitnik *et al.,* 2014). These ‘neprilysin-like’ proteins include: Fra mauro (Frma), which is involved in sex peptide responses and is required for female remating receptivity and fertility (Findlay *et al.,* 2014); Gone early (Goe), which functions in the ovary to limit the number of germline stem cells (Matsuoka *et al.,* 2014); and CG4721, which plays a role in eye development and is also involved in lipid and carbohydrate storage (Nfonsam *et al.,* 2012, Banerjee *et al.,* 2021). While these studies have clearly advanced the current understanding of M13 family functionality in *Drosophila*, the overall physiological relevance of individual members is still far from being understood.

We aimed to generate a comprehensive and up-to-date list of *Drosophila* M13 family members and systematically assess evidence for their expression and functional activity. We applied a combination of literature review and bioinformatic analyses (see Methods) to identify a total of 28 *Drosophila* M13 genes, including two (*CG13283* and *CG4723*) that had not been identified in previous studies (Table 1). These were classified into seven neprilysin (*Nep*) and 21 neprilysin-like (*Nepl*) genes and named accordingly. *Nep* classification required the presence of four conserved sequence motifs in the encoded proteins that are critical to catalytic activity in vertebrate neprilysins: HExxH and ENIAD(xGG) represent zinc-binding domains, CxxW is critical to protein folding and maturation, and NAY/FY mediates substrate or inhibitor binding (Turner *et al.,* 2001, Sitnik *et al.,* 2014). The *Nep* genes comprise the previously named *Nep1*, *Nep2*, *Nep3*, *Nep4* and *Nep5* genes, together with *CG3775* (*Nep6*) and *CG5527* (*Nep7*). The 21 *Nepl*-genes encode proteins that exhibit significant similarity to neprilysins in their primary structure, but lack one or more of the motifs required for catalysis. The symbols of *frma* (*CG3239*) and *goe* (*CG9634*) have not been changed, but *‘Nepl1’* and *‘Nepl2’* have been added as respective synonyms to recognize the fact they were the first and second *Nepl* genes to be characterized. The remaining *Nepl* genes have been given a numerical suffix, incremented based on their genomic location. The revised gene nomenclature has been incorporated into FlyBase (https://flybase.org, Larkin *et al.,* 2021).

It is evident that *Drosophila* has an expanded set of *Nep* and *Nepl* genes compared to mammals (Coates *et al.,* 2000, Bland *et al.,* 2008). The 28 *Drosophila* genes are distributed throughout the genome and are present on all major chromosome scaffolds except chromosome arm 3L (Table 1). Three distinct clusters of *Nepl* genes are evident. *Nepl4*, *Nepl5* and *Nepl6* are located in a 17.6 kb interval (also containing two other genes) at cytological position 26C4-D1 on 2L; *Nepl13*, *Nepl14 and Nepl15* are arranged as tandem repeats in head-to-tail orientation in a 7.4 kb interval at 94C4 on 3R; and *Nepl17*, *Nepl18*, *Nepl19*, *Nepl20* and *Nepl21* are arranged as tandem repeats in a 13.1 kb interval at 98F1 on 3R. These *Nepl* clusters are likely to be the result of local duplication events, consistent with previous phylogenetic analyses (Bland *et al.*, 2008, Sitnik *et al.,* 2014).

We analysed genome-wide RNAseq data (Graveley *et al.,* 2011, Leader *et al.,* 2018) to systematically examine evidence for *Nep* and *Nepl* gene expression. As summarized in Table 1, all 28genes are expressed at some point during development (yellow/red heatmap) and exhibit tissue-specific expression in adults, notably within the brain/CNS, thoracicoabdominal ganglion, fat body, heart and reproductive tracts (green heatmap). For the *Nep* genes, *Nep1-4* are expressed throughout development, with *Nep2* being the most highly expressed during the larval-pupal period, whereas *Nep5-7* are only detected at discrete stages. All *Nep* genes, except *Nep3*, are expressed in the male and/or female reproductive tract, with *Nep2* having particularly high expression in spermathecae. Most *Nep* genes are also expressed in the brain/CNS, while around half show expression in the fat body and/or heart. A similar pattern is seen for the *Nepl* genes: most are expressed at all developmental stages, though expression of *Nepl7* and *Nepl8* is undetectable/extremely low throughout development. *Nepl6* and *Nepl21* are notable for their high expression in prepupae, suggesting an important role during metamorphosis. Most *Nepl* genes are expressed in the spermatheca, while only two (*Nepl3* and *Nepl11*) are detectable in the testis. A major share is also expressed in the fat body and heart, and around half of the *Nepl* genes are expressed in the brain/CNS. Amongst the *Nepl* genes, *frma* is expressed at particularly high levels within the fat body and spermathecae. Overall, these expression data are consistent with previous reports (Thomas *et al.,* 2005, Bland *et al.,* 2007, Iijima-Ando *et al.,* 2008, Meyer *et al.,* 2009, Meyer *et al.,* 2011, Findlay *et al.,* 2014, Matsuoka *et al.,* 2014, Sitnik *et al.,* 2014, Banerjee *et al.,* 2021), and further demonstrate that all 28 *Nep* and *Nepl* genes are actively transcribed and therefore potentially functional, likely acting in a stage- and/or tissue-specific manner.

Finally, we examined Nep/Nepl protein sequences for evidence of a signal peptide or transmembrane domain that would indicate the proteins are secreted or membrane-localized, respectively (see Methods). Remarkably, two Nep and the majority (18) of Nepl proteins lack a predicted transmembrane domain and, instead, possess a predicted signal peptide suggesting that they are secreted (Table 1). Although Nep2 is predicted to possess a transmembrane domain, experimental data indicate that it is secreted, presumably via proteolytic cleavage of a membrane-localized proprotein (Thomas *et al.,* 2005). *Nep4* is unique in encoding either a transmembrane or a secreted isoform as a result of alternative splicing (Meyer *et al.,* 2009), while *Nepl17* encodes a single isoform containing both a signal peptide and a transmembrane domain. One function of the secreted Nepl proteins may be to bind and sequester peptide targets of the catalytically active neprilysins. Such a biological role has already been suggested for Nepl15 (Banerjee *et al.,* 2021) and could establish a novel facet of the neprilysin-mediated regulation of peptide homeostasis.

In summary, we have combined bioinformatic analyses with an evaluation of relevant publications to generate a comprehensive list and classification of genes encoding neprilysin and neprilysin-like proteins in *Drosophila*. The respective dataset has been compiled as a FlyBase gene group report (https://flybase.org/reports/FBgg0000963.html). These resources will support further research and understanding of the biological roles of Nep and Nepl proteins in flies. Identifying the physiological functions and substrates of this set of largely uncharacterized proteins in *Drosophila* may provide clinically relevant insights into neprilysin function in humans.

## Methods

Publications identifying/characterizing *Drosophila* neprilysins were identified using PubMed (https://pubmed.ncbi.nlm.nih.gov) and FlyBase (https://flybase.org, Larkin *et al.,* 2021). Previously published lists of *Drosophila* neprilysins were obtained from Bland *et al.,* 2008, Meyer *et al.,* 2011 and Sitnik *et al.,* 2014. *De novo* identification of *Drosophila* neprilysins was performed using three approaches: (i) searching FlyBase (FB2021_02) for *Drosophila* proteins containing the InterPro signature “Peptidase M13 family (IPR000718)” (Blum *et al.,* 2021); (ii) identifying orthologs of human M13 peptidases (MME (aka NEP), MMEL1, ECE2, ECE1, KEL, PHEX, ECEL1) using the integrative ortholog prediction tool, DIOPT (v8.0) (Hu *et al.,* 2011) via FlyBase; (iii) querying the MEROPS database (release 12.3; https://www.ebi.ac.uk/merops/; Rawlings *et al.,* 2018) for *D. melanogaster* members of the M13 peptidase family. Gene symbol and genomic location data were obtained from FlyBase (FB2021_02). Developmental stage expression data are derived from the modENCODE developmental transcriptome RNAseq dataset (Graveley *et al.,* 2011), as implemented as heatmaps within FlyBase; Table 1 shows the mean expression levels for each major developmental stage. Adult tissue expression data and heatmaps are from FlyAtlas2 (http://flyatlas.gla.ac.uk/FlyAtlas2/index.html; Leader *et al.,* 2018). Male and female tissue-specific data were quantitatively and qualitatively similar, as were data for the brain/CNS and thoracicoabdominal ganglion, and virgin and mated spermathecae; thus, only representative data for female tissues, brain/CNS and mated spermathecae are shown in Table 1. Relatively little *Nep* and *Nepl* expression is seen in the accessory gland or ovary, and so expression in those tissues is not reported in Table 1. Sequence analysis and motif identification was done using TMHMM Server v. 2.0 (http://www.cbs.dtu.dk/services/TMHMM-2.0/), SignalP-5.0 Server (http://www.cbs.dtu.dk/services/SignalP-5.0/) and Motif Scan (https://myhits.sib.swiss/cgi-bin/motif_scan) with all available motif databases being selected. In addition, all sequences were manually analyzed for the presence of respective motifs, combined with sequence alignments (http://multalin.toulouse.inra.fr/multalin/) to ensure proper motif localization.
